# Intermittent Preventive Treatment of Malaria in Pregnancy Coverage Estimates from Population-based Surveys: Reliability of Women's Recall Among Women with ANC Cards

**DOI:** 10.4269/ajtmh.20-1296

**Published:** 2021-06-28

**Authors:** Natasha Hansen, Susan Youll, Lia Florey, Cameron Taylor

**Affiliations:** 1 President’s Malaria Initiative, U.S. Agency for International Development, Washington, District of Columbia;; 2 The Demographic and Health Surveys Program, ICF, Rockville, Maryland

## Abstract

Large household surveys performed to estimate coverage rates for various health interventions, including intermittent preventive treatment, depend on recall. Many studies question the validity of recalled data. Regarding vaccine coverage rates, it is standard practice to validate responses using medical history cards. To validate the coverage rates of intermittent preventive treatment during pregnancy reported by large household surveys, recalled coverage rates were compared with antenatal care card data in Benin, Ghana, Malawi, and Tanzania. The results indicated that recall was comparable to the coverage rates provided indicated by the antenatal care cards. These findings suggest that intermittent preventive treatment coverage rates reported by large household surveys performed using recalled data are valid.

Demographic and Health Surveys (DHS) and Malaria Indicator Surveys (MIS) are large, nationally representative household surveys conducted every 3 to 5 years.^1^ These surveys rely on the participants’ ability to accurately recall receiving health services, including insecticide-treated net (ITN) use, intermittent preventive treatment during pregnancy (IPTp), and the vaccination history of children. The World Health Organization (WHO) recommends IPTp with sulphadoxine-pyrimethamine for pregnant women residing in most sub-Saharan African countries to protect them from malaria during pregnancy.^2^ IPTp is administered monthly during the second and third trimesters of pregnancy during routine antenatal care (ANC) visits; at least three doses of IPTp are recommended during pregnancy.^2^ Key indicators for assessing optimal IPTp coverage are the percentage of women receiving at least one dose (IPTp1+) and the percentage of women receiving at least three doses (IPTp3+).

IPTp coverage is a self-reported indicator in national household surveys; therefore, many of the studies of IPTp uptake cite recall bias as a potential limitation.^3^^–^^5^ The risk of recall bias may lead to inaccurate estimates of coverage. To eliminate recall bias for vaccination coverage rates, it is standard practice in DHS surveys to verify a parent’s recall of their children’s vaccination history using the children’s medical card.^6^ However, this practice is not standardly used to assess interventions that protect pregnant women from malaria. Recall bias is a real concern for IPTp estimates because the data used to calculate IPTp coverage come from questions asked of women who experienced a live birth within the 2 years before completing the survey. This means it could have been as long as 2.5 years since some women received IPTp during the second trimester. Respondents’ health records, such as an antenatal care (ANC) card kept by women during their pregnancies, may be used as a second verification source to increase confidence in self-reported statistics; this practice is similar to the process used for self-reported immunization data in household surveys.^7^

This analysis assessed the validity of self-reported IPTp coverage in nationally representative household surveys by comparing interviewee responses based on recall with the interviewee’s written ANC card record. Several questions and a process of cross-referencing ANC cards were added to the 2017 Malawi MIS, the 2017–2018 Benin DHS, the 2011–2012 Tanzania HIV and Malaria Indicator Survey (THMIS), and the 2014 Ghana DHS.^1^ Surveyors recorded the results of each woman’s recall and what was recorded on her ANC card using the survey questionnaire tool.

We compared the self-reported results to the ANC card data by testing specificity, sensitivity, kappa statistics, and area under the curve (AUC) of the receiver-operating characteristic (ROC) curve for each of these surveys assuming the IPTp frequency reported on the ANC card was the gold standard. The sensitivity represents the likelihood of a woman correctly reporting that she received IPTp during her most recent pregnancy. The specificity represents the likelihood of a woman correctly reporting that she did not receive IPTp. The kappa test was used to assess the overall agreement between the recall and the ANC card data. These statistics were calculated separately for each incidence of IPTp (one dose, IPTp1; two doses, IPTp2; and three or more doses, IPTp3+). For the purpose of this analysis, only women who had experienced live birth within the past 2 years before the survey, attended an ANC appointment at least once, received at least one dose of IPTp, and had an ANC card that was observed by the surveyor were included. Of the eligible women participating in the Benin survey, 69.2% presented an ANC card (*N* = 1,847); 46.8% (*N* = 873) participating in the Ghana DHS, 69.9% (*N* = 658) participating in the Malawi survey, and 47.3% (*N* = 997) participating in the Tanzania survey presented an ANC card. Table 1 shows the IPTp coverage according to background and sociodemographic characteristics of the women.

**Table 1:** Self-reported IPTp coverage by background characteristics and by survey

	1 dose of SP		2 doses of SP		3+ doses of SP		
	% (CI)	*P* value	% (CI)	*P* value	% (CI)	*P* value	n
Benin DHS 2017–2018							
Residence		0.071		0.727		0.114	
Urban	28.6 (25.3–32.2)		41.9 (38.7–45.1)		29.5 (26.4–32.9)		1,108
Rural	32.9 (30.0–35.9)		41.1 (38.2–44.1)		26 (23.3–28.9)		1,562
Mother’s education		0.001		0.003		< 0.0001	
No education	34.3 (31.5–37.2)		43.3 (40.4–46.3)		22.3 (20.0–24.9)		15,33
Primary	28.2 (24.1–32.7)		43.0 (38.8–47.7)		28.7 (25.1–32.7)		561
Secondary or more	25.3 (21.6–29.5)		34.7 (30.7–39.0)		39.9 (35.7–44.3)		576
Wealth quintiles		< 0.0001		0.124		< 0.0001	
Lowest	37.7 (32.2–43.4)		45.7 (40.4–51.0)		16.6 (13.0–21.0)		387
Second	35.8 (31.6–40.4)		44.8 (40.2–49.5)		19.3 (15.9–23.3)		524
Middle	32.5 (27.9–37.4)		39.4 (35.0–43.9)		28.1 (24.1–32.5)		580
Fourth	30.6 (26.5–35)		38.9 (34.5–43.4)		30.6 (26.5–35.0)		602
Highest	21.5 (18.0–25.5)		40.2 (36.1–44.4)		38.3 (33.9–42.8)		577
Age, years		0.545		0.838		0.301	
15–19	33.7 (26.6–41.5)		40.5 (33.0–48.5)		25.8 (19.6–33.2)		192
20–29	30.2 (27.6–33)		41.0 (38.3–43.8)		28.7 (26.1–31.5)		1,486
30–49	31.9 (28.7–35.3)		42.2 (38.9–45.6)		25.9 (23.1–29.0)		991
Time since delivery		0.054		0.94		0.066	
0–12 months postpartum	29.6 (27.0–32.4)		41.5 (38.8–44.2)		28.9 (26.3–31.6)		1,586
13–24 months postpartum	33.3 (30.2–36.4)		41.3 (38.2–44.6)		25.4 (22.6–28.5)		1,085
ANC card presented		0.321		0.925		0.367	
No	29.6 (26.3–33.1)		41.6 (37.7–45.5)		28.8 (25.4–32.5)		823
Yes^1^	31.8 (29.1–34.6)		41.3 (38.8–43.9)		26.9 (24.5–29.5)		1,847
Total	31.1 (28.9–33.4)		41.4 (39.3–43.6)		27.5 (25.4–29.6)		2,670
Ghana DHS 2014							
Residence		0.65		0.041		0.049	
Urban	17.6 (14.0–21.8)		31.8 (27.9–35.9)		50.7 (45.4–56.0)		836
Rural	18.7 (15.9–21.9)		37.8 (33.8–42.0)		43.4 (38.7–48.3)		1,033
Mother’s education		0.773		0.086		0.043	
No education	19.2 (15.5–23.5)		36.5 (32.2–41.1)		44.2 (38.8–49.8)		472
Primary	18.7 (14.7–23.4)		39.9 (33.7–46.4)		41.5 (35.3–47.9)		352
Secondary or more	17.6 (14.4–21.3)		32.9 (29.1–36.9)		49.5 (44.9–54.2)		1,043
Wealth quintiles		0.007		0.071		0.016	
Lowest	17.3 (13.1–22.5)		36.0 (31.0–41.2)		46.8 (39.9–53.8)		406
Second	15.1 (11.3–19.8)		41.7 (34.0–49.8)		43.2 (35.6–51.2)		396
Middle	24.6 (19.2–31.1)		35.1 (28.9–41.7)		42.5 (34.5–50.8)		369
Fourth	21.6 (16.4–27.9)		35.1 (28.9–41.7)		43.4 (26.5–50.4)		258
Highest	12.3 (8.3–17.9)		28.9 (23.4–35.1)		58.7 (52.2–65.0)		339
Age, years		0.147		0.115		0.709	
15–19	20.4 (12.8–30.9)		29.2 (18.4–43.0)		50.4 (35.4–65.3)		120
20–29	20.3 (16.6–24.6)		32.6 (28.9–36.4)		47.1 (42.9–51.4)		877
30–49	15.7 (13.0–19.0)		38.5 (34.0–43.2)		45.7 (41.7–49.8)		871
Time since delivery		0.049		0.437		0.014	
0–12 months postpartum	20.3 (17.4–23.5)		36.1 (32.0–40.3)		43.7 (39.4–48.1)		1,007
13–24 months postpartum	15.8 (12.6–19.6)		34.0 (30.4–37.8)		50.2 (45.5–54.9)		860
ANC card presented		0.552		0.474		0.782	
No	17.5 (14.4–21.1)		36.2 (32.5–40.1)		46.3 (41.9–50.7)		994
Yes^1^	19.0 (15.7–22.8)		33.9 (29.3–38.9)		47.1 (42.1–52.2)		873
Total	18.2 (15.9–20.7)		35.1 (32.2–38.2)		46.7 (43.0–50.4)		1,867
Malawi MIS 2017							
Residence		0.947		0.232		0.262	
Urban	16.9 (13.1–21.7)		42.3 (36.0–48.8)		40.8 (34.1–47.8)		144
Rural	16.8 (13.3–20.9)		37.7 (33.7–41.8)		45.6 (40.9–50.3)		797
Mother’s education		0.541		0.711		0.913	
No education	20.5 (12.5–31.9)		36.5 (28.6–45.2)		43.0 (31.2–55.5)		141
Primary	16.5 (12.7–21.1)		38.1 (33.8–42.7)		45.4 (40.5–50.4)		630
Secondary or more	14.8 (10.4–20.5)		40.8 (34.3–47.7)		44.4 (35.4–52.8)		170
Wealth quintiles		0.582		0.453		0.199	
Lowest	19.4 (12.0–29.7)		39.4 (30.1–49.6)		41.2 (32.3–50.6)		242
Second	18 (11.3–27.6)		32.7 (24.5–42.1)		49.2 (39.4–59.2)		198
Middle	12.4 (7.6–19.5)		35.2 (26.8–44.6)		52.5 (43.3–61.4)		179
Fourth	18.3 (12.1–26.8)		43.2 (34.2–52.7)		38.4 (29.3–48.4)		165
Highest	14.6 (10.5–19.8)		42.4 (35.4–49.7)		43.1 (36.2–50.2)		158
Age, years		0.05		0.143		0.186	
15–19	24.7 (17.0–34.4)		31.1 (24.4–41)		43.2 (34.1–52.7)		160
20–29	14.3 (10.8–18.6)		37.8 (33.0–42.8)		48.0 (42.7–53.5)		515
30–49	16.9 (11.9–23.5)		43.3 (36.2–50.6)		39.8 (32.8–47.3)		265
Time since delivery		0.428		0.512		0.971	
0–12 months postpartum	18.0 (13.9–23.0)		37.2 (32.4–2.3)		44.8 (39.7–50.0)		509
13–24 months postpartum	15.4 (11.3–20.6)		39.7 (34.4–45.3)		44.9 (39.5–50.4)		432
ANC card presented		0.043		0.182		0.002	
No	22.1 (16.5–28.8)		42.3 (36.0–48.8)		35.7 (29.5–42.4)		283
Yes^1^	14.5 (10.9–19.0)		36.7 (32.3–41.4)		48.8 (43.8–53.8)		658
Total	16.8 (13.8–20.3)		38.4 (34.8–42.0)		44.8 (40.7–49.0)		941
Tanzania THMIS 2011–2012							
Residence		0.842		0.81		0.291	
Urban	46.9 (41.0–52.9)		47.8 (41.5–54.1)		5.3 (3.5–8.1)		469
Rural	46.2 (42.6–49.7)		46.9 (43.6–50.3)		6.9 (5.5–8.7)		1,637
Mother’s education		0.206		0.496		0.325	
No education	45.5 (39.0–52.0)		49.1 (42.9–55.3)		5.5 (3.1–9.7)		385
Primary	47.7 (44.2–51.1)		46.0 (42.5–49.6)		6.3 (5.0–7.9)		1,450
Secondary or more	40.5 (33.4–48.1)		49.9 (42.8–57.1)		9.5 (5.2–16.8)		272
Wealth quintiles		0.541		0.816		0.835	
Lowest	48.3 (41.5–55.1)		45.7 (40.0–51.5)		6.1 (3.5–10.2)		398
Second	48.5 (42.6–54.5)		45.2 (38.8–51.8)		6.2 (4.0–9.7)		452
Middle	44.6 (38.7–50.6)		48.0 (42.1–54.1)		7.4 (4.8–11.3)		379
Fourth	47.8 (42.1–53.4)		46.7 (41.0–52.5)		5.5 (2.5–8.7)		451
Highest	42.2 (35.3–49.5)		50.0 (42.7–57.3)		7.8 (4.8–12.5)		426
Age, years		0.577		0.781		0.538	
15–19	49.8 (40.6–59.1)		46.0 (36.8–55.5)		4.2 (2.0–8.6)		200
20–29	46.8 (43.1–50.7)		46.3 (42.8–49.9)		6.8 (5.1–9.1)		1,073
30–49	44.8 (40.1–49.7)		48.4 (43.4–53.4)		6.8 (4.7–9.8)		833
Time since delivery		0.037		0.01		0.531	
0–12 months postpartum	49.4 (45.2–53.7)		43.6 (39.8–47.4)		7.0 (5.3–9.2)		1,098
13–24 months postpartum	42.9 (38.6–47.4)		50.9 (46.7–55.2)		6.1 (4.6–8.2)		1,009
ANC card presented		0.904		0.163		0.001	
No	46.1 (41.9–50.4)		44.9 (40.7–49.2)		9.0 (7.0–11.4)		1,110
Yes^1^	46.5 (42.1–51.0)		49.6 (45.1–54)		3.9 (2.6–5.9)		997
Total	46.3 (43.3–49.4)		47.1 (44.2–50.0)		6.6 (5.4–8.0)		2,106

ANC = antenatal care; CI = confidence interval; DHS = Demographic and Health Survey; IPTp = intermittent preventive treatment during pregnancy; MIS = Malaria Indicator Survey; SP = sulphadoxine-pyrimethamine; THMIS = Tanzania HIV and Malaria Indicator Survey.

In the Benin 2017–2018 MIS and the Ghana 2014 DHS, the proportion of women with IPTp3+ was higher among the more educated and wealthier women. The proportion of women with IPTp3+ was also higher among women in urban settings than among those in rural settings who participated in the Ghana 2014 DHS. The proportions of women with IPTp3+ appeared to be more equitable among women who participated in the Malawi 2017 MIS and the Tanzania 2011–2012 MIS.

For the women who participated in the Malawi 2017 MIS, IPTp3+ coverage was higher among women who presented an ANC card compared with women who did not present an ANC card (49% versus 36%); however, IPTp1 coverage was lower among women who presented an ANC card (15% versus 22%). For the women who participated in the Tanzania 2011–2012 THMIS, IPTp3+ coverage was lower among women who presented an ANC card (4% versus 9%). For the women who participated in the Ghana and Benin surveys, no significant difference was apparent in IPTp coverage based on the ANC card availability.

The proportions of women who received IPTp1, IPTp2, and IPTp3+ according to self-report, ANC card data, both self-report and ANC card data, and the survey are shown in Table 2; however, these data are restricted to the subpopulation who presented ANC cards at the time of the interview. The IPTp3+ coverage was highest for women who participated in the Ghana and Malawi surveys and ranged from 47% to 55% and from 45% to 49%, respectively, depending on method of reporting information. The proportion of women with IPTp3+ in Benin ranged from 23% to 28%. However, the IPTp3+ coverage was lowest in Tanzania (range, 2–5%).

**Table 2:** IPTp rates reported by women who presented ANC cards^1^

		% (CI)	Total
Benin DHS 2017–2018			
Percentage who received IPTp1 during ANC	Self-report	31.8 (29.1–34.6)	1,847
	ANC card	31.2 (28.5–34.1)	
	Either source	35.6 (32.8–38.5)	
Percentage who received IPTp2 during ANC	Self-report	41.3 (38.8–43.9)	
	ANC card	39.9 (37.4–42.4)	
	Either source	44.6 (42.1–47.2)	
Percentage who received IPTp3+ during ANC	Self-report	26.9 (24.5–29.5)	
	ANC card	23.2 (20.9–25.7)	
	Either source	27.6 (25.2–30.2)	
Ghana DHS 2014			
Percentage who received IPTp1 during ANC	Self-report	19 (15.7–22.8)	873
	ANC card	15.5 (12.6–18.9)	
	Either source	21.1 (17.7–25)	
Percentage who received IPTp2 during ANC	Self-report	33.9 (29.2–38.9)	
	ANC card	30.8 (27–34.8)	
	Either source	39.9 (35.2–44.8)	
Percentage who received IPTp3+ during ANC	Self-report	47.1 (42.1–52.2)	
	ANC card	47.5 (43.1–51.9)	
	Either source	54.7 (49.9–59.4)	
Malawi MIS 2017			
Percentage who received IPTp1 during ANC	Self-report	14.5 (10.9–19)	658
	ANC card	19.1 (15.6–23.3)	
	Either source	19.6 (16–23.7)	
Percentage who received IPTp2 during ANC	Self-report	36.7 (32.3–41.4)	
	ANC card	35.6 (31–40.5)	
	Either source	38.7 (34.1–43.5)	
Percentage who received IPTp3+ during ANC	Self-report	48.8 (43.8–53.8)	
	ANC card	44.5 (39.8–49.2)	
	Either source	48.8 (43.8–53.8)	
Tanzania THMIS 2011–2012			
Percentage who received IPTp1 during ANC	Self-report	46.5 (42.1–51)	997
	ANC card	43.7 (39.6–47.8)	
	Either source	50.6 (46.2–55)	
Percentage who received IPTp2 during ANC	Self-report	49.6 (45.1–54)	
	ANC card	47.4 (43.3–51.5)	
	Either source	54.1 (49.7–58.4)	
Percentage who received IPTp3+ during ANC	Self-report	3.9 (2.6–5.9)	
	ANC card	2.1 (1.2–3.4)	
	Either source	4.8 (3.2–7.1)	

ANC = antenatal care; CI = confidence interval; DHS = Demographic and Health Survey; IPTp = intermittent preventive treatment during pregnancy; MIS = Malaria Indicator Survey; THMIS = Tanzania HIV and Malaria Indicator Survey.

To test the validity of women’s recall of IPTp doses received, we calculated the sensitivity and specificity of IPTp1, IPTp2, and IPTp3+ by comparing self-report with ANC card data. Additionally, we calculated a kappa statistic to test the agreement between the two measures and the AUC of the ROC curve to test the individual validity of the self-report measure compared with that of the ANC card data (Table 3).

**Table 3:** Sensitivity, specificity, and agreement of IPTp coverage comparing self-report with ANC card records among women who presented an ANC card

	Sensitivity	Specificity	ROC curve area	
	% (CI)	% (CI)	% (CI)	Kappa statistic
Benin DHS 2017–2018				
Recall of IPTp1	88.4 (85.4–91)	94 (92.6–95.3)	0.912 (0.9–0.93)	0.8162
Recall of IPTp2	91.7 (89.4–93.6)	92.3 (90.5–93.8)	0.92 (0.91–0.93)	0.8377
Recall of IPTp3	97.2 (95.1–98.5)	94.4 (93–95.5)	0.958 (0.95–0.97)	0.8655
Ghana DHS 2014				
Recall of IPTp1	82.5 (75.3–88.4)	92.4 (90.2–94.2)	0.874 (0.84–0.91)	0.6887
Recall of IPTp2	79.3 (74–84)	86.6 (83.6–89.2)	0.83 (0.8–0.86)	0.6431
Recall of IPTp3	84 (80.1–87.5)	86.4 (82.9–89.3)	0.852 (0.83–0.88)	0.7039
Malawi MIS 2017				
Recall of IPTp1	79.1 (70–86.6)	99.6 (98.6–100)	0.894 (0.85–0.93)	0.8526
Recall of IPTp2	95.6 (92–97.9)	96.4 (94–98)	0.96 (0.94–0.98)	0.9164
Recall of IPTp3	100 (98.7–100)	94.3 (91.2–96.5)	0.971 (0.96–0.98)	0.938
Tanzania THMIS 2011–2012				
Recall of IPTp1	91.6 (88.8–94)	87.5 (84.5–90.1)	0.896 (0.88–0.91)	0.7871
Recall of IPTp2	91.2 (88.3–93.6)	88.4 (85.4–90.9)	0.898 (0.88–0,92)	0.7928
Recall of IPTp3	72.7 (49.8–89.3)	97.8 (96.7–98.6)	0.853 (0.76–0.95)	0.5203

ANC = antenatal care; CI = confidence interval; DHS = Demographic and Health Survey; IPTp = intermittent preventive treatment during pregnancy; MIS = Malaria Indicator Survey; ROC = receiver-operating characteristic; THMIS = Tanzania HIV and Malaria Indicator Survey.

Across surveys, the sensitivity of women's ability to correctly report IPTp coverage ranged from 79% to 92% for IPTp1, from 79% to 96% for IPTp2, and from 73% to 100% for IPTp3+. The proportion of women who correctly reported that they did not receive IPTp (specificity) ranged from 88% to 99.6% for IPTp1, from 87% to 96% for IPTp2, and from 83% to 98% for IPTp3+. The agreement between self-reported IPTp coverage and coverage indicated on ANC cards, as measured by kappa scores, ranged from 0.69 to 0.85 for IPTp1, from 0.64 to 0.92 for IPTp2, and from 0.52 to 0.94 for IPTp3+. Although interpreting kappa scores can be somewhat problematic, the scores found during this analysis represent a moderate to high level of agreement on most scales.^8^^,^^9^ The AUC of the ROC curve values were > 0.8 for all reported IPTp measurements from all surveys and > 0.9 for the majority, indicating an excellent to outstanding level of individual validity for the self-reported coverage estimates.^10^^,^^11^

This analysis demonstrates that self-reported recall of IPTp is an adequate method of reporting IPTp with sulphadoxine-pyrimethamine doses received by women who had experienced a live birth within the 2 years before the survey using the standard protocols for household surveys such as the DHS and MIS. The analysis suggests that additional validation through external sources, such as ANC cards and a review of medical records, is not necessary. It has been found that using local context (i.e., a local word for iron folate) or a visual aid (photograph of a drug) can improve recall.^12^^,^^13^ For example, because of the potentially long period of recall (up to 2.5 years), the accuracy of IPTp recall may be improved by providing visuals of sulphadoxine-pyrimethamine tablets during household survey interviews to help women recall the number of IPTp doses they received.

This study only includes data from four countries; therefore, the results presented here may not be representative of all countries implementing IPTp with different rates of coverage. Another limitation of this study was that the dataset was restricted to include only women who were able to present their ANC card to the interviewer; therefore, only 47% of the women in Ghana and Tanzania, 69% in Benin, and 70% in Malawi were eligible for this study. This group of women may be different than the group women who were unable or unwilling to present their ANC card at the time of the survey. Additional studies that use health facility records of IPTp administration to verify self-reported data could be considered to address this bias. Furthermore, the surveys used for this study covered a wide date range and a wide range of IPTp coverage. The Tanzania survey was conducted in 2011 to 2012, but the others were conducted more recently. The IPTp3+ coverage indicated by this early Tanzania survey was low; therefore, these data could have affected the sensitivity and agreement statistics. Finally, the potential for interviewer bias should be considered because it is possible that during the interviews, surveyors reconciled self-reported responses based on the data observed on the ANC card.

Despite these limitations, after validation using the ANC card data as the gold standard, the results of this study demonstrate that the self-reported IPTp coverage estimates indicated by these national household surveys are accurate. The results of this study support the validity of recall of IPTp indicated by large household surveys, thus suggesting that national malaria control programs can reliably assess their IPTp intervention coverage and make programmatic decisions based on survey data. Additionally, this analysis demonstrates that including additional questions and ANC card data verification may not be necessary and is not likely to significantly change IPTp coverage estimates.
